# Early diagnosis of atherosclerosis with panoramic radiographs: a review

**DOI:** 10.1590/1677-5449.002316

**Published:** 2016

**Authors:** Daiane Landim Borba, Ulisses Vilela Hipólito, Yamba Carla Lara Pereira

**Affiliations:** 1 Faculdade de Ciências do Tocantins – FACIT, Curso de Odontologia, Araguaína, TO, Brazil.

**Keywords:** panoramic radiographs, atherosclerosis, early diagnosis, radiografia panorâmica, aterosclerose, diagnóstico precoce

## Abstract

Carotid artery disease has been linked with cerebral vascular accident, also known as stroke, cerebral hemorrhage, or cerebral ischemia. It is caused by narrowing or obstruction of arteries in the neck (the carotid arteries) that are responsible for transporting blood from the aorta to the brain. Panoramic radiographs are used in dentistry to show both dental arches as a supplement to the clinical dental examination. The objective of this study is to highlight the importance of panoramic radiographs for diagnosis of arterial disease, by means of a bibliographic review. The PubMed database was searched using the keywords “atherosclerosis” and “panoramic”, with the filters “last 5 years” and “humans”. Twenty articles were identified, six of which were chosen for this study because they were open access. The review concluded that panoramic radiographs enable early diagnosis of carotid artery calcification, resulting in earlier interventions, and offer an accessible cost.

## Resumo

Doenças das artérias carótidas estão associadas a casos de acidente vascular cerebral, também chamados de derrame ou isquemia cerebral. Ocorrem devido ao estreitamento ou obstrução das artérias do pescoço, ou artérias carótidas, responsáveis por levar o sangue da aorta para o cérebro. A radiografia panorâmica é um exame de ambos os arcos dentários que ajuda no exame clínico odontológico. Destacar a importância da radiografia panorâmica no diagnóstico de doença arterial é o objetivo deste trabalho, que foi realizado através de pesquisa bibliográfica na PubMed, com as palavras-chave “atherosclerosis” e “panoramic”, e os filtros: últimos 5 anos e humanos. Vinte artigos foram elencados, dos quais seis formaram a base para este estudo, graças ao acesso livre ao seu conteúdo. Conclui-se que a radiografia panorâmica permite o diagnóstico precoce de calcificação da artéria carótida, levando a uma intervenção precoce a um custo acessível.

## INTRODUCTION

Cerebrovascular accidents (strokes) are the third greatest cause of death in industrialized countries. Atherosclerosis is a pathology that is related to strokes and consists of formation calcium-rich fatty plaques on the walls of arteries and their ramifications, which can be diffuse and/or localized and contribute to narrowing and hardening of the arteries, in combination with accumulation of fat in the artery walls, which is known as atheroma.^1^

They are generally seen radiographically in individuals over the age of 50 and affect both sexes.^2^ Carotid artery disease can cause cerebrovascular and encephalic accidents (also known as strokes or cerebral ischemia), since they are directly associated with narrowing or blockage of the arteries in the neck (carotid arteries) that carry blood from the aorta to the brain.^3^

In many cases, the disease is related to traditional risk factors, such as high blood pressure, diabetes, high cholesterol, smoking, and obesity, in which the symptoms only appear when blood vessels are almost completely blocked.^4^ Atherosclerosis has chronic inflammatory characteristics and can cause death and disability, making it a serious public health problem because of the high cost of efforts to rehabilitate patients.^5^ Of the different methods to diagnose atherosclerotic disease, angiography is considered the “gold standard”.^6^ However, since 1981, certain radiopaque images seen in panoramic dental X-rays have been described as a sign of the presence of calcified carotid atheroma plaques.^7^ Panoramic radiography provides images of the middle third of the face, obtained using an extra-oral technique in which the X-ray machine rotates around the patient, acquiring a virtual image.^8^ This technique enables examination of both dental arches and the neighboring structures in a single X-ray. Their practicality and comprehensiveness means they are considered the diagnostic examination of choice, in combination with the clinical examination.^9^

In asymptomatic individuals who are at risk of stroke, atherosclerosis of the carotid artery (ACA) can be identified in a panoramic radiograph as a diffuse bilateral radiopaque image extending from the region of the ramus and angle of the mandible to the base of the neck.^5^ This examination therefore has an important role to play in early diagnosis of ACA.^10^ When ACA is diagnosed, atherosclerosis treatment can be initiated by the appropriate professional, with the objective of repairing or mitigating acute or chronic ischemic lesions, thereby preventing serious manifestations and preserving the patient’s quality of life.^11^

In 2006, Friedlander assessed 94 people (mean age of 65.6 years) for the presence of atheromatous plaques using panoramic radiographs and in 50% of them atheromatous plaques were found in carotid arteries.^12^ This subset had occult metabolic syndrome, which comprises a combination of abdominal obesity, elevated triglycerides, reduced HDL levels, high blood pressure, insulin resistance, and atheroma plaques.^12^

The common carotid artery ascends through the mid-cervical area, where it bifurcates giving rise to the external and internal carotid arteries.^13^ The location of this bifurcation varies slightly and, on rare occasions, may occur so far below the normal level that it is no longer visible on a panoramic radiograph.^14^ Therefore, a risk of stroke may go undetected in such patients if this type of imaging examination is employed.^5^ However, if the region between cervical vertebrae C3 and C4 is observed carefully, and structures located in the same region are differentiated, it is possible to detect signs of atherosclerosis in the carotid arteries using panoramic radiographs, thereby anticipating treatment and reducing patient morbidity and mortality.^15^

Detection of atheroma plaques in the carotid artery by examination with panoramic radiographs is valuable for early diagnosis and for minimizing patient risk, contributing to early referral of patients for treatment.

## MATERIAL AND METHOD

This is a review of bibliography identified by searching the PubMed Web site with the keywords: “atherosclerosis” and “panoramic”. The filters used were “last 5 years” and “humans”.

## RESULT

**Figure 1:**
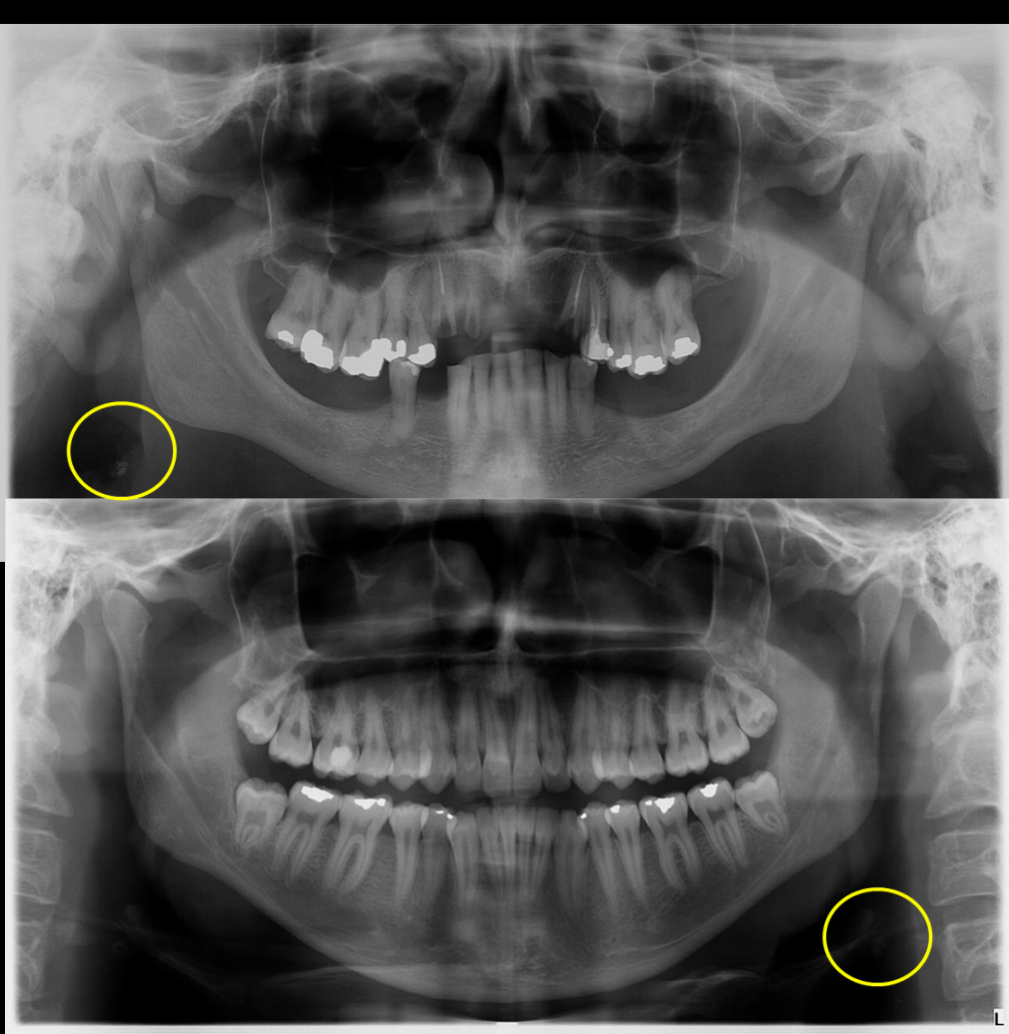
Images compatible with carotid atherosclerotic lesion detected by panoramic radiography.

The search results identified 20 articles, 6 of which were reviewed for this study, chosen because they were open access. Four publications were from 2013 and two were from 2012 (Table 1 and Figure 1).

**Table 1:** Descriptive table of items found in the database, with authors and year of publication.

**Article**		**Author**	**Date**
1	Diagonal ear lobe crease and atherosclerosis: A review of the medical literature and dental implications.	Arthur H Friedlander et al.^16^	2012
2	Prevalence of calcified carotid artery atheromas on panoramic images of individuals with primary hyperparathyroidism.	Arthur H Friedlander et al.^17^	2013
3	Recognizing Calcifications of the Carotid Artery on Panoramic Radiographs to Prevent Strokes.	Sonja Baumann-Bhalla et al.^18^	2012
4	Structural evidence of anti-atherogenic microRNAs.	Anthony Virtue et al.^19^	2011
5	The prevalence and correlation of carotid artery calcification on panoramic radiographs and peripheral arterial disease in a population from the Republic of Korea: the Dong-gu study.	J-S Lee et al.^20^	2013
6	Validation of a method for quantifying carotid artery calcification from panoramic radiographs.	Amy C Alman et al.^21^	2013

## DISCUSSION

Atherosclerosis is one of the leading causes of mortality in Brazil.^22^ It is a chronic inflammatory disease of multifactorial origin, in which there is accumulation of fat, cholesterol, and other substances on the artery walls, restricting blood flow and causing many different health problems.^23^ Factors that contribute to the emergence of arterial disease include: high cholesterol, diabetes, obesity, smoking, family history of heart disease, physical inactivity, chronic kidney disease, and others.^24^

One of the manifestations of atherosclerosis is formation of atheroma, which are characterized by large accumulations of lipids, fibrous tissues, calcium deposits, blood, and blood products, among others.^25^ Plaque formation occurs after endothelial damage followed by tissue repair.^26^ The risk factors can damage the vascular endothelium, causing endothelial dysfunction and mediating entry of monocytes, which include modified proteins such as oxidative LDL, for example, causing foam cells.^27^ Inflammatory mediators are released, extending the process and forming the plaques.^28^ Cerebrovascular accidents (strokes), also known as encephalic vascular accidents,^29^ are one of the leading causes of death and disability all over the world.^30^ They are characterized by blood leakage in brain tissues.^31^ When blood circulation is interrupted, functional and structural changes also occur in the region involved, establishing a complex “ischemic cascade”, the final result of which is neuronal death.^32^ In these cases, the diagnosis of the disease is based on the patient's clinical status associated with a neurological examination.^33^

The first X-ray was performed on November 8, 1895, by Rontgen, who used the left hand of his wife, Anna Bertha Rontgen, placed between a frame holding photographic film and a cathode ray tube that emitted radiation for 15 minutes. After the film was developed, the shape of his wife’s hand was revealed, showing the bones inside less dense soft tissue.^34^ Radiographic examinations are of fundamental importance in dentistry because they supplement the clinical examination and are essential for diagnosis and for planning and monitoring dental treatments.^35^ Dentistry relies on two different types of X-ray techniques: intra-oral and extra-oral. In intra-oral methods, the X-ray film or sensor is placed inside the patient's mouth. This category in turn can be subdivided into periapical, occlusal, and interproximal, which aim to provide detailed views of dental elements and adjacent bone tissue.^36^ Extra-oral techniques consist of taking X-rays with the radiographic film or sensor exposed outside of the patient's mouth.^37^ The most widely used of the extra-oral techniques is panoramic radiography, which offers visualization of both dental arches and their surrounding structures in a single X-ray.^38^ There are both analog and digital methods.^39^

Panoramic radiography is of fundamental importance in early and incidental diagnosis of carotid atheroma.^40^ It is the dental surgeon’s duty to recognize atherosclerosis when inspecting panoramic radiography images and to instruct patients to seek confirmation with other examination methods and to refer them to the relevant health professional for appropriate treatment.^41^ This is in order to ensure patients’ quality of life and well-being.^42^

In a 2012 article, Friedlander et al. discussed the need to identify atherosclerosis and its risk factors before the complications set in. They stated that atherosclerosis is the leading cause of deaths in Spain and drew an analogy of the prevalence of the disease to extra-vascular signs related to earlobe creases.^16^ They emphasize that such creases are incipient signs of circulatory problems in the head and neck area and that their etiology is associated with atherosclerotic disease. They recommended that, although further research is advisable, dentists should conduct examinations of their patients’ ears, checking for diagonal earlobe creases and, in conjunction with clinical history, vital signs, and panoramic radiographs, decide on the need for a medical assessment of the patient.^16^ In 2013, the same group of researchers presented reports associating primary hyperparathyroidism (PHPT) to carotid complications. They stated that calcified carotid artery atheroma are often recorded in panoramic images of patients with PHPT and so health professionals should be vigilant.^17^

Baumann-Bhalla et al. consider that it is important to examine more closely the panoramic radiographs that are taken every day in Switzerland, especially in relation to arterial calcification, and to direct affected patients to a specialist in order to confirm or rule out this diagnosis. They stressed that on panoramic radiographs, it not only teeth and jaws that should be analyzed, but also the areas at the sides of the image, especially in patients over the age of 50 and in patients with risk factors, thereby making early recognition of calcifications more likely and preventing cerebrovascular events.^18^

Virtue et al.^19^ have studied regulation of pro-inflammatory genes influencing inhibition of production of miRNAs with inflammatory potential. Their study suggests a treatment avenue, based on an understanding of the protective mechanism provided by miRNAs, especially through suppression of the atherogenic effects of certain genes, and also shows that individual patient characteristics are risk factors for triggering atherosclerotic processes.

Data published in 2013 illustrate the prevalence of carotid artery calcification (CAC), detected with panoramic radiographs and associated with peripheral arterial disease (PAD). They analyzed the difference in PAD prevalence between patients with and patients without CAC detectable on panoramic radiographs. The study sample comprised 4078 subjects aged 50 or older (1410 males and 2668 females) who had undergone medical and dental examinations in the city of Gwangju, South Korea. Panoramic radiographs and presence of carotid artery calcification were analyzed. Presence of PAD was determined by measuring the ankle-brachial index (ABI). An ABI of 0.9 in any leg was considered evidence of PAD. The prevalence of CAC in panoramic radiographs was 6.2 and the PAD prevalence was 2.6, in middle-aged or older patients. It is known that it is important to detect CAC and peripheral artery disease to prevent fatal events such as ischemic stroke and myocardial infarction.^20^

Digital panoramic radiographs were used to evaluate the area of carotid artery calcification using tools available in ImageJ, while inpatient and outpatient discharge records were reviewed to identify patients who had undergone Doppler ultrasound examination of the carotid arteries. Area under the curve analysis showed that quantification of carotid artery calcification correlated well with degree of stenosis. They concluded that quantification of carotid artery calcification using digital panoramic images can identify patients who need further evaluation, such as with conventional medical tests.^21^

## CONCLUSION

It is concluded that panoramic radiography, a routine examination used by dentists, offers the opportunity for early diagnosis of carotid artery calcification, enabling interventions while disorders are still incipient, ensuring preservation of patients' quality of life at an accessible cost, accomplished using an inexpensive imaging exam that is widely available for dentists, neurologists, and angiologists.
